# A Case of Abortion, Brought on by Savin, and Followed by Perforation of the Stomach, and Death

**Published:** 1851-06

**Authors:** 


*A Case of Abortion brought on by Savin, and followed by Perforation of the Stomach, and Death.* Communicated by James H. Salisbury M. D., of Albany, to T. R. Beck, M. D.— A lady by the name of Miss ——— came into Albany at 3 o'clock on Wednesday morning, July 24th, 1850, on the western train of cars, and stopped at tne Delavan House. She entered her name on the register ——, and took a room on the lower floor. About 9 o'clock A. M. the same day, she requested a room on the second floor, where she would not be so much disturbed by the noise from the street. At the same time she stated that she wished to remain a few days. Her countenance was pale and careworn—like one sick and troubled. Room 44 on the second floor was given her.

On Wednesday afternoon about 4 o'clock, she requested one of the waiters to go and get some medicine for her in a two ounce phial, labeled, chloroform. She told him to get the medicine that was written on the label of the phial. This was obtained for her, as requested, at Dr. Burton's, near the Delavan House. On the following morning, about 5½ A. M., she rang the bell, and requested the waiter to go to a drug store and get some medicine. She gave him the two-ounce phial, labeled *chloroform*, and told him to get it filled with the medicine written on the label, and also to get a drachm phial of morphine. This the waiter did as requested. No more was heard or seen of her until Friday, 2 P. M., July 26.

At 2 P. M., Friday, July 26th, the key to the door was found on the outside in the hall on the floor, and the door bolted on the inside. On looking through the keyhole, a portion of a lady's wardrobe was observed lying on a chair. The door was immediately burst open by Mr. Clark, assisted by Mr. Colburn.

The body of the deceased lay upon the bed in an easy and natural position, apparently dead. The coroner (Mr. Brower) was immediately called to hold an inquest. Dr. Swinburn was summoned to make a post-mortem. He called on me to assist him.

*Autops. cadav.* about thirty-three hours after she was last seen alive, and probably about twelve or fourteen hours after death. She was not yet cold. She lay on her back in the bed in an easy and natural position, her left hand lying over the region of the stomach and her right hand lying off to the right of her at an angle of about 45°. Near her right hand lay two phials, the one containing chloroform, the other morphine.

Her appearance, externally, was normal, except some white froth which had issued, and was still issuing from her nose. On removing her from the bed, found her underclothes and the sheets considerably stained with blood in the region of the hips. The blood afterwards was found to have proceeded from the uterus. On laying open the abdomen, found the stomach bearing marks of high inflammation. It was softened and perforated, and its contents emptied into the cavity of the abdomen. There was extensive peritonitis. The perforation was about the size of a fifty cent piece, and was situated in the region of the greater curvature, near the cardiac orifice. For several inches around the perforation, the stomach was very much corroded, thinned, and softened, so that it was easily torn. Œsophagus in a state which indicated high inflammation. Small intestines very much inflamed for about four feet from the stomach, the remaining portion comparatively healthy. The colon and rectum blackened and inflamed in the vicinity of scybala, which were found to the amount of about a pint scattered through their whole length. These were carefully removed and preserved, together with the intestines, stomach, and œsophagus, for chemical examination. Uterus was found enlarged. It had the appearance of a recently evacuated gravid uterus of from three to four months gone. Empty, except about two ounces of lochial discharge or secretion. Mouth of uterus relaxed and open, so as to admit easily the finger. Vulva and vagina loose and flabby. Judging from the state of the parts, I should think the fœtus had been discharged from two to three days.

She appeared to be about twenty-seven years of age, of a moderately full habit, and possessing naturally a strong constitution.

*Chemical Examination.*—The stomach, intestines, and their contents, with about one quart of matter vomited up in the chamber, consisting mostly of tea and coffee, and the several bottles containing medicine found in her possession, were delivered to me for chemical examination. This examination was immediately commenced. From the corroded appearance and perforation of the stomach, the presence of some corrosive mineral poison was suspected. After subjecting the stomach and intestines, the scybala, the contents of the chamber, and what matter had been emptied from the stomach into the cavity of the abdomen, separately to a rigid chemical examination, for all of those mineral poisons which would be likely to produce such a state of things in the stomach, without finding the slightest evidence of any of them, I commenced the search of each part separately for the several vegetable substances of an irritating nature, used as emmenagogues. The first substance tested for was savin. Slight evidence of its presence was found in the stomach and intestines, still greater evidence of its presence in the matter vomited, and that taken from the abdominal cavity, and conclusive evidence of its presence in the scybala, or hardened feces. The examination of the aforesaid parts of the body here ended.

Attention was next directed to the contents of the several phials found in her possession. The only one suspected to contain savin was examined. There was about half a drachm in the phial. It was made up of a mixture of oil of savin and tinct. of lavender. No other substance was found in any of the phials which would be at all likely to irritate and perforate the stomach. Here the examination for poisons ended. Of the drachm of morphine which she obtained Thursday morning, only thirty grains were left. Of the two ounces of chloroform, only about one half ounce remained. So that from

Thursday morning to the time of her death, which probably occurred Thursday night, she swallowed, or otherwise disposed of thirty grains of morphine and one and a half ounces of chloroform.

From papers in her trunk, she appeared to be unmarried. The state she was in showed conclusively that she had been pregnant, and between three and four months gone; from the chemical examination, that savin had been administered; from the circumstances of the case, that it had been given or taken to produce an abortion; from the post mortem, that a violent gastritis had been excited, which resulted in abortion, softening and perforation of the stomach, peritonitis and death.—*Am. Jour. of Medical Sciences.*

